# Skeletal muscle radiodensity as an automated survival predictor in men with metastatic castration-resistant prostate cancer undergoing PSMA-targeted radioligand therapy

**DOI:** 10.1007/s00259-026-08068-x

**Published:** 2026-07-22

**Authors:** Christian Immanuel, Thomas Büttner, Alois M. Sprinkart, Sebastian Nowak, Maike Theis, Jim Küppers, Sibgol Fazeli, Leander Fritzsche, Aland Amin, Dilan Salih, Hojjat Ahmadzadehfar, Florian C. Gärtner, Barbara Kreppel, Rupert Conrad, Manuel Ritter, Markus Essler, Philipp Krausewitz, Milka Marinova

**Affiliations:** 1 Department of Nuclear Medicine, University Hospital Bonn, Venusberg Campus 1, 53127 Bonn, Germany; 2 Department of Urology and Pediatric Urology, University Hospital Bonn, Bonn, Germany; 3 Department of Diagnostic and Interventional Radiology, University Hospital Bonn, Bonn, Germany; 4 Department of Nuclear Medicine, Institute of Radiology, Neuroradiology and Nuclear Medicine, Knappschaft Kliniken, University Hospital Bochum, Bochum, Germany; 5 Department of Nuclear Medicine, Knappschaft Kliniken Westfalen Dortmund, Dortmund, Germany; 6 Department of Psychosomatic Medicine and Psychotherapy, University Hospital Muenster, Muenster, Germany

**Keywords:** Castration-Resistant Prostate Cancer, Lutetium-177, PSMA, Skeletal muscle radiodensity, Survival predictor

## Abstract

**Purpose:**

Skeletal muscle radiodensity (SMD) is an emerging imaging-derived marker of muscle quality. Its prognostic relevance in patients with metastatic castration-resistant prostate cancer (mCRPC) undergoing [¹⁷⁷Lu]Lu-PSMA-617 radioligand therapy has not yet been systematically evaluated. This study aims to investigate the prognostic value of SMD derived from routine pretherapeutic PET/CT imaging in a large real-world mCRPC cohort treated with [¹⁷⁷Lu]Lu-PSMA-617.

**Methods:**

In this single-center study, the largest to date cohort comprising 410 mCRPC patients was analyzed. SMD was quantified automatically from the L3-level CT component of pretherapeutic [⁶⁸Ga]Ga-PSMA-PET/CT using an AI-based segmentation tool. An optimal SMD cut-off (19.26 HU) was determined for survival stratification. Associations with overall survival (OS) were assessed using multivariable Cox regression (univariable and multivariable, adjusted for baseline PSA) and Kaplan-Meier analysis.

**Results:**

Lower SMD was associated with reduced OS (HR 1.87, *P*<.001). Median OS were significantly shorter in the SMD-low group compared to the SMD-high group (7.6 months vs. 12.2 months; *P*<.001). This prognostic stratification was seen in both, patients with or without PSA50 or PSA90 response, respectively. SMD correlated with age, albumin, and PSA, but not with BMI.

**Conclusion:**

SMD assessed from routine pretherapeutic PET/CT is a non-invasive prognostic biomarker in patients undergoing PSMA-targeted radioligand therapy. Integration of host-derived imaging biomarkers such as SMD with tumor-specific parameters may improve risk stratification, prehabilitation and support future individualized treatment strategies in advanced prostate cancer.

## Introduction

Metastatic castration-resistant prostate cancer (mCRPC) is an advanced form of cancer characterized by progressive growth and complex, challenging management, with a continuing demand for effective treatment options. In recent years, [^177^Lu]Lutetium vipivotide tetraxetan prostate-specific membrane antigen (PSMA) therapy ([^177^Lu]Lu-PSMA-617) has emerged as a promising targeted treatment [1–3]. By binding to PSMA expressed by mCRPC cells, this complex is internalized by the tumor cells and accumulates in cancer tissue, inducing antitumoral toxicity via β^−^-emission. Following the positive results of the phase-3 VISION trial, [^177^Lu]Lu-PSMA-617 was approved by both the European Medicine Agency (EMA) and the U. S. Food and Drug Administration (FDA) in 2020 for the treatment of mCRPC after progression on second-generation antiandrogens and chemotherapy. In patients with mCRPC treated with radioligand therapy following progression on prior systemic therapy, median overall survival (OS) of approximately 12–18 months and radiographic progression-free survival (rPFS) ranging from 4 to 9 months have been reported, depending on study design and patient selection. Treatment choices should balance efficacy with toxicity and quality of life (QoL), making individualized prognosis prediction an ongoing priority [4].

Owing to the theranostic character of [¹⁷⁷Lu]Lu-PSMA-617 –intergating both diagnostics and targeted radioligand therapy– pre-treatment imaging is a mandatory prerequisite for patient selection. Baseline [^68^Ga]Ga-PSMA-positron emission tomography combined with computed tomography ([^68^Ga]Ga-PSMA-PET/CT) to assess PSMA avidity of metastatic lesions was an inclusion criteria into the VISION trial and has become the standard of care [3]. This imaging modality provides prognostic information, with PSMA uptake in metastatic lesions serving as a predictor of OS [5–7].

With the implementation of the Prostate Cancer Molecular Imaging Standardized Evaluation (PROMISE) criteria, PSMA-PET has demonstrated prognostic utility across various stages of the disease [7]. However, tracer uptake analyses have limitations even when assessed semi-automatically. Thus, the concomitant CT component offers an opportunity to extract additional host-related predictive markers.

Current prognostic assessment primarily relies on tumor-derived imaging biomarkers such as PSMA uptake intensity, total tumor volume (TTV), and clinical parameters including performance status and prior lines of therapy. While these approaches capture tumor burden and biological activity, they incompletely reflect host-related physiologic reserve, which may also influence treatment tolerance and survival within a theranostic paradigm.

CT-based body composition analysis has emerged as an important tool, as sarcopenia and myosteatosis are increasingly recognized predictors of adverse outcomes across oncologic and non-oncologic diseases [8]. Skeletal muscle radiodensity (SMD) provides a quantitative surrogate for muscle quality, with lower SMD linked to inferior survival in several advanced malignancies, including lung, colorectal, and renal cell carcinoma [9–12]. Recent advances in artificial intelligence (AI) enable fully automated extraction of SMD from routine imaging [13, 14].

Building on proof-of-concept reports suggesting prognostic value of body composition [15, 16], we hypothesized that AI-derived SMD represents an independent host-related prognostic biomarker in patients with mCRPC undergoing PSMA-targeted radioligand therapy and provides complementary prognostic information beyond established tumor-specific and biochemical response markers. Accordingly, we aimed to evaluate the clinical prognostic utility of AI-derived SMD in a large real-world cohort of mCRPC patients treated with [¹⁷⁷Lu]Lu-PSMA-617.

## Materials and methods

### Patient cohort and ethics

**Fig. 1:**
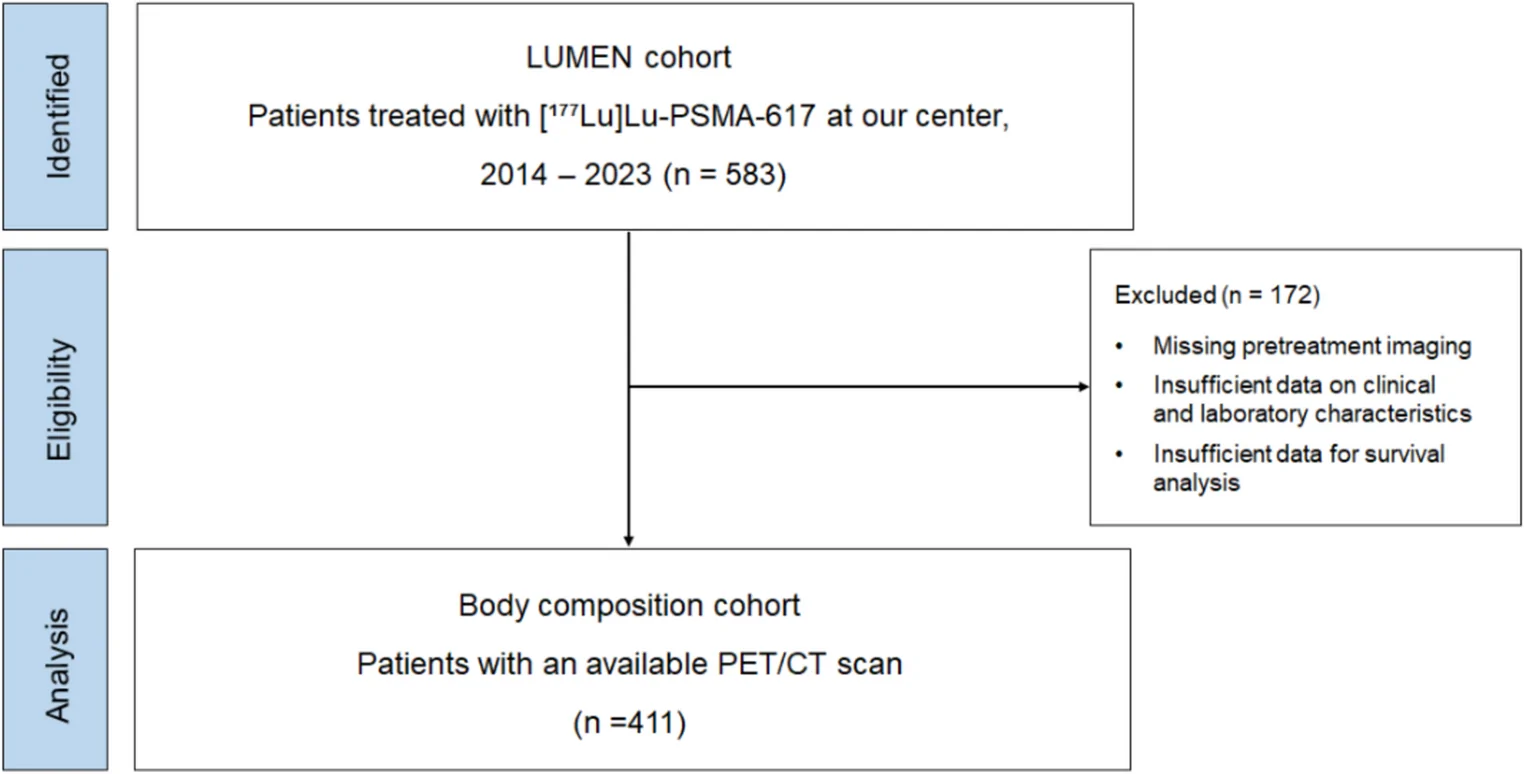
Consort diagram of the study PET/CT: positron emission tomography/computed tomography

**Fig. 2:**
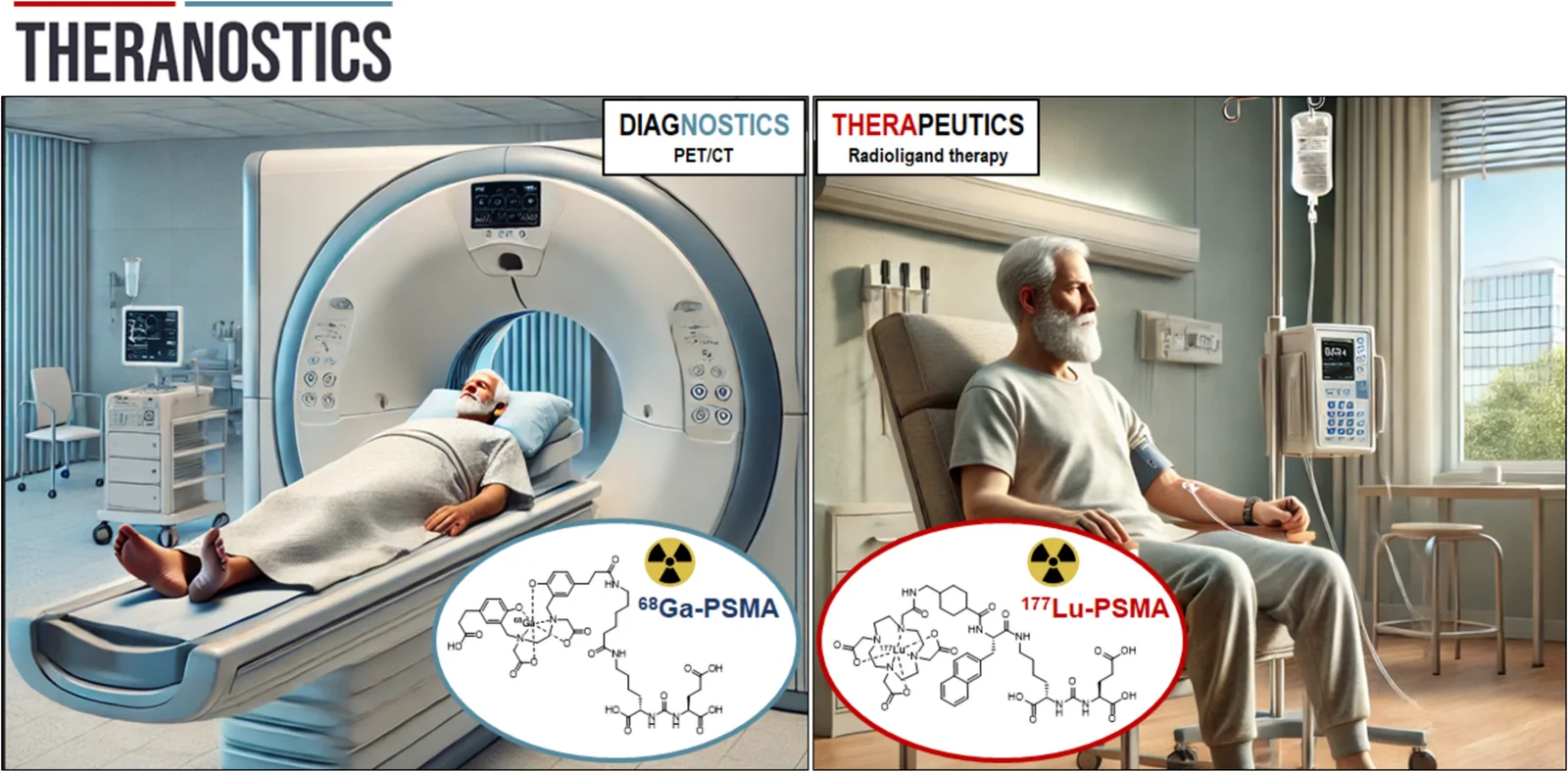
Schematic illustration of theranostics in prostate cancer: diagnostic [⁶⁸Ga]Ga-PSMA PET/CT enables detection and staging of PSMA-expressing lesions, whereas therapeutic [¹⁷⁷Lu]Lu-PSMA delivers targeted radioligand therapy to the same molecular target (PET/CT: positron emission tomography/computed tomography).

The study (German Clinical Trial Register: DRKS00035087) was approved by the institutional review board (Ethics Committee vote 2024-19-BO) and conducted in accordance with the Declaration of Helsinki, with a waiver of informed consent because of the retrospective study design. It included mCRPC patients who received at least one cycle of [^177^Lu]Lu-PSMA-617 therapy at our institution, between 2015 and the data cut-off in December 2023 (Figs. 1 and 2). Inclusion criteria comprised histologically confirmed mCRPC, receipt of at least one cycle of [¹⁷⁷Lu]Lu-PSMA-617 therapy at our institution, and availability of pretherapeutic baseline [⁶⁸Ga]Ga-PSMA-PET/CT imaging suitable for quantitative CT analysis at the L3 level.

### Clinical data

Baseline data included age, Eastern Cooperative Oncology Group (ECOG) performance status, BMI, metastatic burden, and key laboratory parameters. Biochemical response was defined as PSA50 (≥ 50% decline of PSA value) and PSA90 (≥ 90% decline of PSA value) from baseline.

### Imaging

Whole-body PET/CT was performed from the skull base to mid-thigh 30–45 min after intravenous administration of [⁶⁸Ga]Ga-PSMA (2 MBq/kg body weight) using two different scanners depending on the year of imaging. From 2014 to 2021, studies were acquired on a Biograph 2 (BI) PET/CT system (Siemens Medical Solutions). Beginning in 2021, imaging was performed on a Vereos (VE) PET/CT system (Philips Healthcare). PET emission data were acquired 5 min (BI) or 1 min (VE) per bed position. The axial field of view was 15.8 cm (BI) and 16.4 cm (VE). PET data was processed using an iterative reconstruction algorithm with corrections for attenuation, scatter, randoms, and decay. Images were reconstructed with a 512 × 512 matrix and a 60-cm field of view, yielding an in-plane spatial resolution of approximately 4.6 mm (BI) and 3 mm (VE). A low-dose CT, performed immediately before PET acquisition, was used for attenuation correction and anatomical localization (120 kVp; automatic tube current modulation, typically 25–80 mAs; pitch, 0.8–1.2; slice thickness, 1–5 mm; standard soft-tissue and bone kernels).

### Skeletal muscle radiodensity

**Fig. 3:**
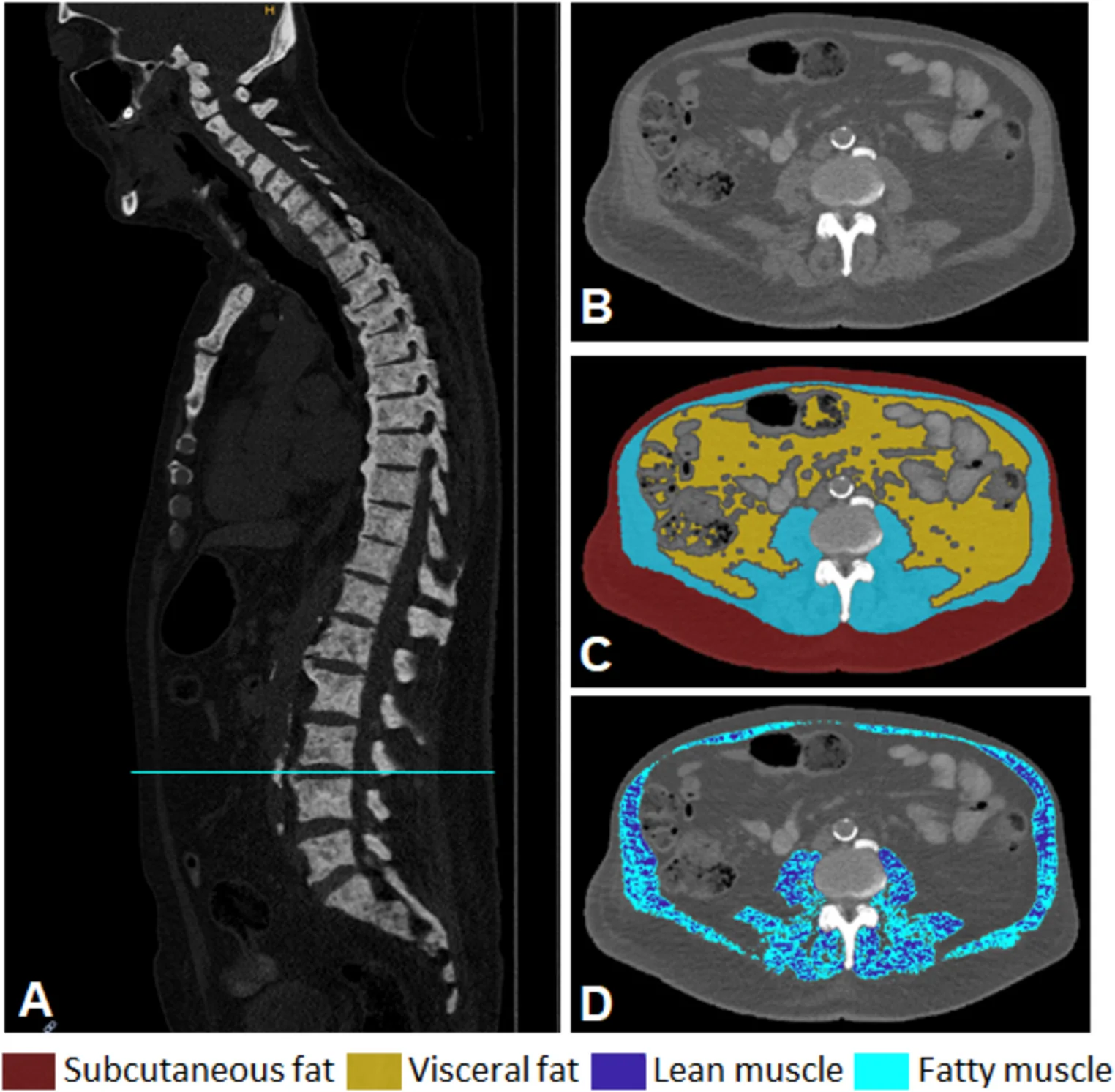
Images of a 75-year old man with metastatic castration-resistant prostate cancer (diagnosed in 09/2018; initial PSA 2706 ng/mL) A. Sagittal CT image in the bone window at the midline showing diffuse osteoblastic bone metastases B. Axial CT image in the soft-tissue window at the level of lumbar spine L3/4 (indicated by the line in image A) C. Body composition analysis of image B using a deep-learning model. The segmented area was classified into different tissue types based on Hounsfield unit ranges (red: subcutaneous adipose tissue; blue: muscle; yellow: visceral adipose tissue) D. Muscle tissue subdivided into lean muscle (dark blue) and fatty muscle (light blue) (CT: computed tomography; PSA: prostate-specific antigen)

SMD was derived from AI-assisted automated analysis of CT scans reconstructed with a soft-tissue kernel at the L3/L4 intervertebral disc space, as previously validated [13–18]. In prior validation studies, the model achieved high agreement with manual ground truth segmentation, with reported Dice similarity coefficients exceeding 0.95 for skeletal muscle compartments. Automated outputs were visually reviewed to ensure anatomical plausibility (Fig. 3). Transverse cross-sections at this level were used for body composition analysis, with SMD calculated as the mean Hounsfield unit within the segmented muscle area. SMD was assessed at three standardized time points: baseline (available for all patients), after three treatment cycles, and at end of therapy, depending on imaging availability.

SMD was selected as the primary quantitative parameter because CT-derived muscle attenuation has been widely validated as a surrogate of myosteatosis and muscle quality, demonstrating reproducibility and prognostic relevance across oncologic populations.

### Endpoints

OS was defined as the time from the first cycle of [¹⁷⁷Lu]Lu-PSMA-617 therapy to death from any cause or last follow-up. OS was compared between SMD-low (< 19.26) and SMD-high (> 19.26) patients in the overall cohort and in stratified subgroups.

### Statistics

Data were presented as means ± standard deviation (SD) or medians with interquartile range, depending on distribution. Group comparison used Welch two sample t-test, Wilcoxon rank sum test, Kruskal-Wallis test, or Chi-square-test as appropriate. Survival hazards were estimated with univariable Cox regression for SMD and multivariable Cox regression including SMD and baseline PSA as the covariates, and survival curves compared using the Kaplan-Meier method and log-rank tests. Furthermore, to formally assess whether the prognostic impact of SMD was independent of biochemical response, interaction terms between continuous SMD and PSA response status (both PSA50 and PSA90) were incorporated into the Cox regression models. To evaluate the robustness and stability of the identified cut-off, a nonparametric bootstrapping procedure with 1,000 resamples was performed. In each bootstrap iteration, the optimal cutpoint was then re-estimated, and the distribution of these estimates was used to calculate bias, standard error, and the 95% confidence interval (CI) based on the percentile method. P-values < 0.05 were considered statistically significant. Analysis was performed in RStudio (v2024.09.0 + 375) using the packages *survival* (v3.8-3), *survminer* (v0.5.0), *maxstat* (v0.7-26), and *ggpubr* (v.0.6.1).

## Results

**Fig. 4:**
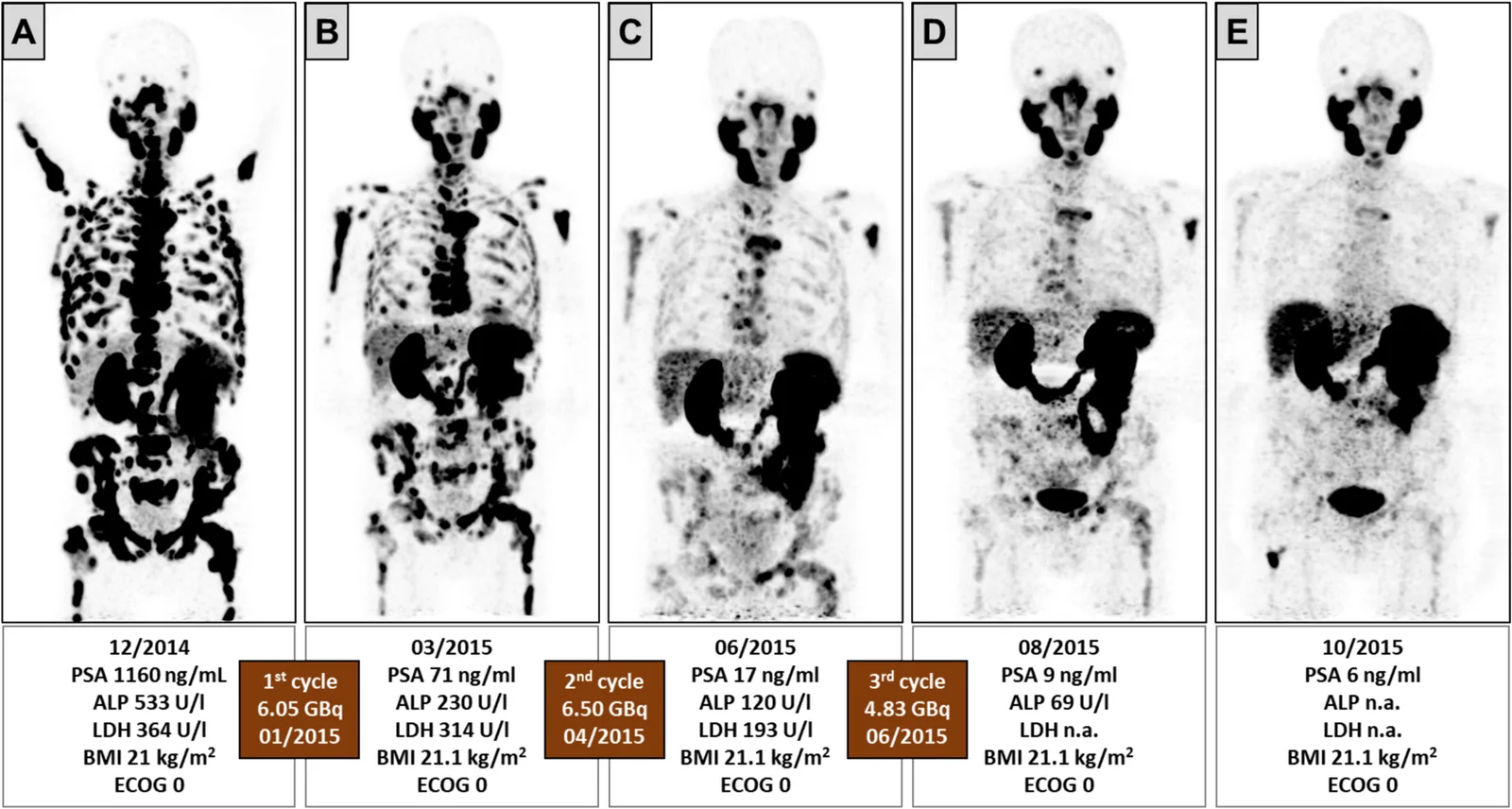
Representative case of a 75-year-old man with metastatic castration-resistant prostate cancer and diffuse bone metastases undergoing [¹⁷⁷Lu]Lu-PSMA radioligand therapy: [⁶⁸Ga]Ga-PSMA PET MIP images **A**. MIP image (coronal plane) obtained prior to radioligand therapy shows extensive diffuse bone metastases (initial PSA level, 1160 ng/mL) **B**–**E**. MIP images (coronal plane) obtained during and after three cycles of [¹⁷⁷Lu]Lu-PSMA radioligand therapy demonstrate an excellent treatment response, accompanied by a marked decrease in PSA level (PSA level, 6 ng/mL at the time of image E) ALP: alkaline phosphatase; BMI: body mass index; ECOG: Eastern Cooperative Oncology Group; LDH: lactate dehydrogenase; MIP: maximum intensity projection; PET: positron emission tomography; PSA: prostate-specific antigen

A total of 410 patients from the LUMEN cohort were included in the analysis (Fig. 1). Baseline characteristics are provided in Table 1. The cohort represented a real-world population with advanced disease, reflected by a median age of 72.7 years and a high median baseline PSA level of 152 ng/mL. A representative case with a good response to PSMA radioligand therapy is shown in Fig. 4. More than half of the patients (58.3%) presented with reduced general condition (ECOG performance status ≥ 1), and 16.9% exhibited poor functional status (ECOG ≥ 2).

**Table 1:** Baseline clinical characteristics as assessed at [^177^Lu]Lu-PSMA-617 therapy start

Parameter	SMD high *n* = 326	SMD low *n* = 84	Overall *n* = 410	*P*-value^1^
Age (years)				< 0.001***
Mean (SD)	71.1 (8.9)	74.9 (6.7)	71.8 (8.6)	
Median (range)	71.7 [39.6, 93.0]	75.0 [61.1, 91.9]	72.7 [39.6, 93.0]	
Body-Mass-Index (kg/cm^2^)				0.613
Mean (SD)	25.9 (4.3)	25.6 (4.1)	25.8 (4.3)	
Median (range)	25.3 [15.6, 47.6]	25.3 [16.4, 35.5]	25.3 [15.6, 47.6]	
Unknown	13 (4.0%)	5 (6.0%)	18 (4.4%)	
ECOG PS (n=, %)				0.004**
0	145 (44.5%)	26 (31.0%)	171 (41.7%)	
1	119 (36.5%)	30 (35.7%)	149 (36.3%)	
2	42 (12.9%)	19 (22.6%)	61 (14.9%)	
3	6 (1.8%)	2 (2.4%)	8 (2.0%)	
Unknown	14 (4.3%)	7 (8.3%)	21 (5.1%)	
Prior therapy (n=, %)				
Prostatectomy	125 (38.3%)	29 (34.5%)	154 (37.6%)	0.742
Prostate irradiation	62 (19.0%)	14 (16.7%)	76 (18.5%)	0.597
1 L chemotherapy	221 (67.8%)	67 (79.8%)	288 (70.2%)	0.044*
2 L chemotherapy	87 (26.7%)	30 (35.7%)	107 (28.5%)	0.134
ARSI	156 (47.9%)	31 (36.9%)	187 (45.6%)	0.28
Metastases (n=, %)				
Lymph nodes				0.440
Pelvic	119 (36.5%)	24 (28.6%)	143 (34.9%)	
Retroperitoneal	51 (15.6%)	13 (15.5%)	64 (15.6%)	
Supradiaphragmatic	134 (41.1%)	42 (50.0%)	176 (42.9%)	
Bone				0.10
< 5	12 (3.7%)	1 (1.2%)	13 (3.2%)	
5–10	22 (6.7%)	3 (3.6%)	25 (6.1%)	
> 10	252 (77.3%)	72 (85.7%)	324 (79.0%)	
Visceral metastases	76 (23.3%)	31 (36.9%)	107 (26.1%)	0.017*
PSA (ng/mL)				0.002**
Mean (SD)	377 (696)	498 (587)	402 (677)	
Median (range)	136 [0.1, 5670]	272 [0.8, 2800]	152 [0.1, 5670]	
Unknown	5 (1.5%)	3 (3.6%)	8 (2.0%)	
SMD (HU)				
Mean (SD)	28.4 (6.0)	14.9 (4.0)	25.6 (7.8)	
Median (range)	27.7	16.0	25.4	
	[19.3, 46.7]	[-1.50, 19.2]	[-1.50, 46.7]	

*1L* one line of chemotherapy, *2L* two lines of chemotherapy, *ALP* alkaline phosphatase, *ARSI* androgen receptor signaling inhibitor, *ECOG* *PS* Eastern Cooperative Oncology Group Performance Status, *mCRPC* metastatic castration-resistant prostate cancer, *PSA* prostate-specific antigen, *SD* standard deviation, *SMD* skeletal muscle radiodensity,

^1^ SMD-low vs. SMD-high, calculated by Welch’s t-test, Wilcoxon test, Kruskal-Wallis test or Chi-square test, as appropriate

*P<.05, **P<.01, ***P<.001

### Skeletal muscle radiodensity

Mean SMD was 25.6 ± 7.8 HU, with a median of 25.4 HU (range, − 1.5 to 46.7). Maximally selected rank statistics identified 19.26 as the optimal cut-off for OS stratifying patients into SMD-high (> 19.26) and SMD-low (< 19.26) groups. Bootstrap validation with 1,000 resamples confirmed the stability of the identified cut-off, demonstrating minimal bias (0.94 HU). The 95% bootstrap confidence interval for the threshold ranged from 16.57 to 26.07 HU, supporting robust estimation of the prognostic cutpoint within this cohort.

Age, ECOG PS, PSA, albumin, hemoglobin, alkaline phosphatase (ALP), visceral metastases, and prior chemotherapy differed significantly between groups, whereas BMI, creatinine, other pretreatments, and metastatic patterns did not (Table 1). Mean SMD declined to 22.4 ± 6.7 HU after three cycles (*P*=.092) and to 22.4 ± 7.0 HU in the last imaging available (*P*=.007).

### Overall survival

In Cox regression analysis, SMD below the cut-off was significantly associated with inferior OS (HR, 1.87; 95% CI: 1.45–2.41; *P*<.001). When analyzed as a continuous variable, decreasing SMD remained associated with increased mortality risk (HR per HU decrease, 1.02; 95% CI: 1.01–1.04; *P*=.003), supporting a biologically graded association rather than a threshold-only effect.

To further validate these findings against a marker of disease burden, a multivariable Cox regression analysis was performed including both continuous SMD and baseline PSA *as the covariates*. In this model, continuous SMD retained its independent prognostic value for OS (HR per HU decrease, 1.02, 95% CI: 1.031–1.001, *P*=.036), demonstrating that its predictive capacity is distinct from tumor burden, as reflected by baseline PSA (HR per ng/mL increase, 1.00, 95% CI: 1.00–1.00; *P*<.001). The detailed results of the multivariable model are summarized in Table 2.

**Table 2:** Multivariable Cox regression analysis for overall survival

Covariate	Hazard ratio (HR)	95% CI	P-value^1^
SMD (per 1 HU decrease)	1.016	1.030 – 1.001	.036*
Baseline PSA (per 1 ng/mL increase)	1.000	1.000 – 1.001	<0.001***

*HR* Hazard Ratio, *CI* Confidence Interval, *SMD* skeletal muscle radiodensity, *PSA* prostate-specific antigen

*P<.05; ***P<.001

**Fig. 5:**
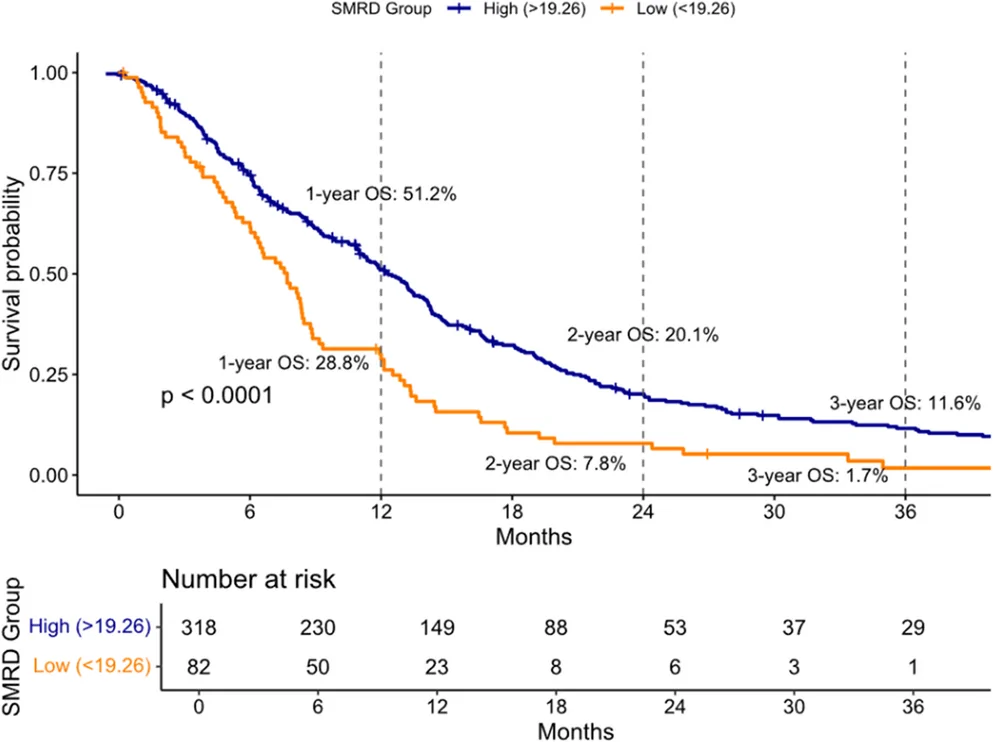
Skeletal muscle radiodensity (SMD, SMRD), stratified into SMD-high and SMD-low using a cut-off value of 19.26 HU, as a predictor of overall survival (OS). Kaplan–Meier analysis demonstrates significantly different overall survival between groups, with 1-, 2-, and 3-year survival rates significantly lower in the SMD-low group (all *P*<.001)

Log-rank analysis confirmed the prognostic value of the SMD cut-off, with a median OS of 7.6 months (95% CI: 6.0–8.5) in the SMD-low group versus 12.2 months (95% CI: 11.0–13.8) in the SMD-high group (*P*<.001). One-, two-, and three-year survival rates were markedly lower in the SMD-low group (28.8%, 7.8%, and 1.7%) compared with the SMD-high group (51.2%, 20.1%, and 11.6%) (Fig. 5). All statistical analyses were conducted at the patient level. Each data point represents one individual patient, with a single SMD measurement derived from a predefined axial CT slice at the L3 vertebral level.

### PSA response subgroups

**Fig. 6:**
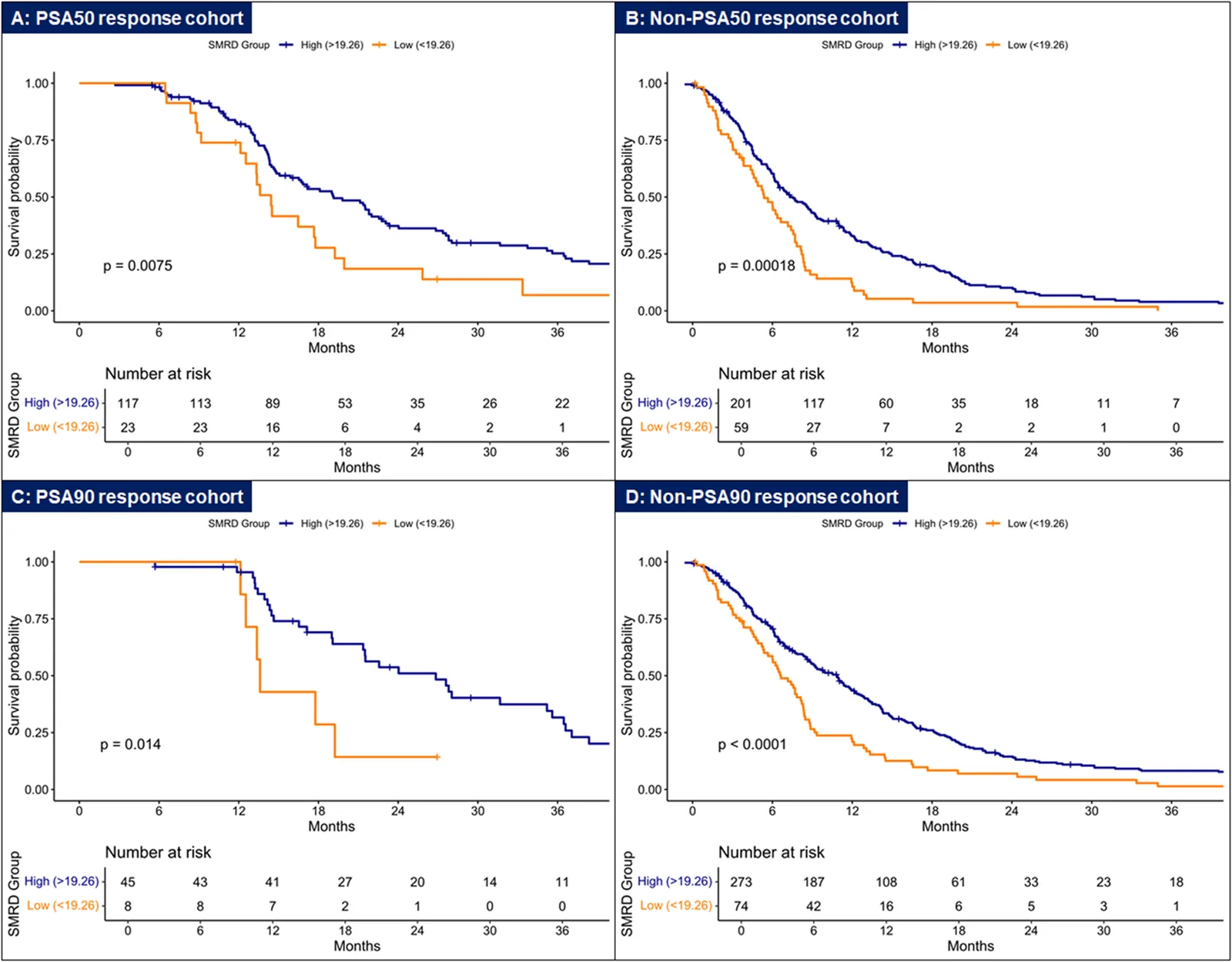
Kaplan–Meier estimates of overall survival stratified by skeletal muscle radiodensity (SMD, SMRD), dichotomized using a cut-off value of 19.26 HU, in patients with a PSA50 response (**A**), without a PSA50 response (**B**), with a PSA90 response (**C**), and without a PSA90 response (**D**). In all groups, SMD was a significant predictor of overall survival.

Subgroup analyses by biochemical response confirmed the prognostic relevance of SMD. In both PSA50 responders and non-responders, as well as in PSA90 responders and non-responders, low SMD remained significantly associated with a shorter OS (all *P*<.001, Fig. 6). Formal interaction testing confirmed that the prognostic impact of SMD on overall survival was independent of the PSA response pattern. In the model assessing PSA50, the main effects for continuous SMD (HR per 1 HU decrease 1.021, 95% CI: 1.038–1.005; *P*=.011) and PSA50 response (HR 0.350, 95% CI: 0.150–0.814; *P*=.015) were both significant, while the interaction term was not statistically significant (HR 1.001, 95% CI: 0.970–1.033; *P*=.953). Similarly, in the model assessing PSA90, continuous SMD retained prognostic significance (HR 1.020, 95% CI: 1.036–1.004; *P*=.012), whereas PSA90 response did not reach statistical significance as a main effect in this specific interaction model (HR 0.403, 95% CI: 0.123–1.319; *P*=.133), and the interaction term for PSA90 remained non-significant (HR 1.001, 95% CI: 0.958–1.046; *P*=.959).

## Discussion

To our knowledge, this is the first study to evaluate the prognostic value of SMD, automatically derived from routine pretherapeutic PET/CT scans, in a large real-world cohort of patients with mCRPC undergoing [¹⁷⁷Lu]Lu-PSMA-617 radioligand therapy. Our findings demonstrate that lower SMD, reflecting impaired muscle quality, is independently associated with inferior OS. Consequently, they support the use of SMD within prehabilitation programs aimed at optimizing patients’ physical condition.

The relevance of [¹⁷⁷Lu]Lu-PSMA-617 therapy in mCRPC was established in the phase III VISION trial, which demonstrated its survival benefit in patients with progressive disease following androgen receptor pathway inhibition and chemotherapy [3]. While VISION focused primarily on treatment efficacy and tumor-specific imaging criteria, our study addresses a complementary dimension—namely the prognostic relevance of host-derived imaging markers such as muscle quality. Pretherapeutic [⁶⁸Ga]Ga-PSMA-PET/CT imaging—required for VISION eligibility and now standard of care—provides a unique opportunity to extract additional prognostic information through body composition analysis using the same imaging dataset.

Our findings suggest that survival heterogeneity under PSMA-targeted radioligand therapy is influenced not only by tumor burden but also by host-related physiologic reserve. Opportunistic extraction of SMD from routine PSMA-PET/CT may therefore complement established tumor-based biomarkers without additional imaging burden. While not yet practice-changing, SMD could contribute to future integrated prognostic models combining tumor, treatment, and host-related factors.

Host-related factors such as sarcopenia and muscle quality have increasingly been recognized as relevant prognostic indicators across oncologic disciplines [19–23]. In particular, previous studies in cardiovascular and oncologic patients have linked low skeletal muscle quality with frailty, systemic inflammation, and diminished treatment tolerance [24–27]. Our results confirm and extend two earlier proof-of-concept reports that suggested a prognostic role of body composition metrics in mCRPC [15, 16]. Using a large cohort and a fully automated, AI-driven workflow, we validate these initial findings and demonstrate the feasibility of incorporating muscle analysis into clinical imaging pipelines. An optimal SMD cut-off enabled robust risk stratification. Patients below this threshold showed significantly shorter survival and higher mortality rates, independent of ECOG performance status and BMI. Importantly, this association was not reflected in early biochemical response rates (PSA50 or PSA90), suggesting that SMD captures host vulnerability beyond classical tumor or performance markers. These findings align with previous reports demonstrating that sarcopenia—defined by low muscle mass, density, and function—predicts not only survival but also radiographic progression and treatment-related toxicity in patients with mCRPC receiving androgen receptor signaling inhibitors (ARSIs) or chemotherapy [28, 29]. Moreover, longitudinal analyses indicate that bone-targeted therapies such as Radium-223 do not accelerate sarcopenia, supporting the notion that muscle loss primarily reflects disease biology and androgen deprivation [30]. Our study extends these observations to PSMA-targeted therapy in mCRPC, a setting in which muscle quality has not been systematically evaluated.

As previously shown, sarcopenic patients with mCRPC undergoing either ARSI or chemotherapy experienced more frequent radiographic progression (HR 2.39), higher overall mortality (HR 2.44), and a greater incidence of grade ≥ 3 treatment-related toxicity (OR 3.27), underscoring the prognostic and functional burden of muscle degradation in this population [28].

Our data confirm and extend the role of body composition analysis as an opportunistic, non-invasive marker derived from standard-of-care imaging. Unlike BMI or ECOG, SMD reflects intrinsic muscle characteristics such as fat infiltration (myosteatosis), which are not captured in routine assessment. SMD has been linked to inflammation, nutritional status, and even immune competence—all factors with potential relevance for systemic therapy response and tolerance [23–26, 31, 32].

The lack of association between SMD and biochemical response (PSA decline) supports its role as a complementary prognostic dimension reflecting systemic resilience rather than tumor-specific activity. Crucially, our multivariable analysis confirmed this complementary relationship by demonstrating that SMD retains its independent prognostic value for overall survival even when directly adjusting for baseline PSA. This indicates that poor muscle quality independently contributes to mortality risk regardless of the underlying disease burden. In contrast, biochemical markers capture the tumor-specific response, but do not reflect the patient’s physiologic reserve—an aspect that may be better characterized by SMD. A comparable dissociation between muscle quality and tumor marker response was also described previously when sarcopenia independently predicted shorter failure-free survival (HR 6.69) and earlier PSA progression (HR 12.91) in patients with metastatic hormone-sensitive prostate cancer treated with docetaxel or abiraterone, despite similar PSA response rates [33]. These observations may indicate that low muscle quality predicts survival independent of tumor burden or procedural risk.

From a methodological perspective, the use of AI-driven, reproducible muscle analysis ensures scalability and objectivity—two key prerequisites for clinical translation. As demonstrated in prior studies, automated pipelines can reduce reader variability, streamline workflow, and improve reproducibility across institutions [34–36]. Importantly, CT-derived muscle assessment requires no additional imaging or patient burden, facilitating its rapid integration into routine clinical practice.

Although CT attenuation values are expressed in HUs, variations in scanner calibration, tube voltage, reconstruction kernel, and slice thickness may influence absolute SMD measurements. Consequently, the cut-off identified in this single-center cohort should be interpreted cautiously and validated externally before broader application. In addition, SMD reflects muscle quality (myosteatosis) rather than the full diagnostic spectrum of sarcopenia, which also includes muscle mass and functional performance. Therefore, SMD should be considered a surrogate marker of muscle integrity rather than a comprehensive frailty assessment.

This study has some limitations. Its retrospective and single-center design may introduce bias, and although our cohort represents the largest to date in this context, prospective multi-center validation is warranted. The single-center design and use of institution-specific imaging protocols may further limit generalizability, underscoring the need for external validation in multicenter cohorts with harmonized acquisition parameters. Although SMD demonstrated independent prognostic value, its role in clinical decision-making should currently be regarded as complementary rather than practice-changing. Integration into structured reporting systems and multimodal risk models may support future translation into individualized treatment strategies. Residual confounding and the need for protocol harmonization and workflow integration should be considered before broader clinical implementation.

## Conclusion

SMD derived from routine pretherapeutic PSMA-PET/CT represents a promising host-related prognostic biomarker in patients with mCRPC undergoing [¹⁷⁷Lu]Lu-PSMA-617 radioligand therapy. As an opportunistically available parameter, SMD may complement established clinical and tumor-based metrics by providing additional prognostic information beyond tumor response or performance status. Prospective multicenter validation and integration with established tumor-specific markers are required before incorporation into routine clinical decision-making.

## Data Availability

The datasets generated during and/or analyzed during the current study are available from the corresponding author on reasonable request.

## References

[1] Kratochwil C, et al. [177Lu]Lutetium-labelled PSMA ligand-induced remission in a patient with metastatic prostate cancer. Eur J Nucl Med Mol Imaging. 2015;42(6):987–8. 10.1007/s00259-014-2978-1 25573634

[2] Afshar-Oromieh A, et al. The Theranostic PSMA Ligand PSMA-617 in the Diagnosis of Prostate Cancer by PET/CT: Biodistribution in Humans, Radiation Dosimetry, and First Evaluation of Tumor Lesions. J Nucl Med. 2015;56(11):1697–705. 10.2967/jnumed.115.161299 26294298

[3] Sartor O, et al. Lutetium-177–PSMA-617 for Metastatic Castration-Resistant Prostate Cancer. N Engl J Med. 2021;385(12):1091–103. 10.1056/NEJMoa2107322 PMC8446332 34161051

[4] Patell K, et al. Lutetium-177 PSMA for the treatment of metastatic castrate resistant prostate cancer: a systematic review. Expert Rev Anticancer Ther. 2023;23(7):731–44. 10.1080/14737140.2023.2213892 37194261

[5] Seifert R, et al. Semiautomatically Quantified Tumor Volume Using < sup>68 Ga-PSMA-11 PET as a Biomarker for Survival in Patients with Advanced Prostate Cancer. J Nucl Med. 2020;61(12):1786–92. 10.2967/jnumed.120.242057 32332147

[6] Seifert R, et al. Analysis of PSMA expression and outcome in patients with advanced Prostate Cancer receiving < sup>177 Lu-PSMA-617 Radioligand Therapy. Theranostics. 2020;10(17):7812–20. 10.7150/thno.47251 PMC7359095 32685021

[7] Karpinski MJ, et al. Combining PSMA-PET and PROMISE to re-define disease stage and risk in patients with prostate cancer: a multicentre retrospective study. Lancet Oncol. 2024;25(9):1188–201. 10.1016/S1470-2045(24)00326-7 39089299

[8] Cruz-Jentoft AJ, Sayer AA. Sarcopenia Lancet. 2019;393(10191):2636–46. 10.1016/S0140-6736(19)31138-9 31171417

[9] Bradshaw PT. Body composition and cancer survival: a narrative review. Br J Cancer. 2024;130(2):176–83. 10.1038/s41416-023-02470-0 PMC10803330 37891197

[10] Sjøblom B, et al. Skeletal muscle radiodensity is prognostic for survival in patients with advanced non-small cell lung cancer. Clin Nutr. 2016;35(6):1386–93. 10.1016/j.clnu.2016.03.010 27102408

[11] Kroenke CH, et al. Muscle radiodensity and mortality in patients with colorectal cancer. Cancer. 2018;124(14):3008–15. 10.1002/cncr.31405 PMC6033621 29797673

[12] Vrieling A, et al. Body Composition in Relation to Clinical Outcomes in Renal Cell Cancer: A Systematic Review and Meta-analysis. Eur Urol Focus. 2018;4(3):420–34. 10.1016/j.euf.2016.11.009 28753824

[13] Nowak S, et al. Deep learning–based assessment of CT markers of sarcopenia and myosteatosis for outcome assessment in patients with advanced pancreatic cancer after high-intensity focused ultrasound treatment. Eur Radiol. 2024;34(1):279–86. 10.1007/s00330-023-09974-6 PMC10791981 37572195

[14] Nowak S, et al. Fully Automated Segmentation of Connective Tissue Compartments for CT-Based Body Composition Analysis: A Deep Learning Approach. Invest Radiol. 2020;55(6):357–66. 10.1097/RLI.0000000000000647 32369318

[15] Hartrampf PE, et al. Elevated Body Mass Index Is Associated with Improved Overall Survival in Castration-Resistant Prostate Cancer Patients Undergoing Prostate-Specific Membrane Antigen–Directed Radioligand Therapy. J Nucl Med. 2023;64(8):1272–8. 10.2967/jnumed.122.265379 37290794

[16] Roll W, et al. Prognostic value of body composition out of PSMA-PET/CT in prostate cancer patients undergoing PSMA-therapy. European Journal of Nuclear Medicine and Molecular Imaging; 2025. 10.1007/s00259-025-07416-7 PMC12660389 40580320

[17] Faron A, et al. CT fatty muscle fraction as a new parameter for muscle quality assessment predicts outcome in venovenous extracorporeal membrane oxygenation. Sci Rep. 2020;10(1):22391. 10.1038/s41598-020-79495-5 PMC7769972 33372188

[18] Nowak S, et al. End-to-end automated body composition analyses with integrated quality control for opportunistic assessment of sarcopenia in CT. Eur Radiol. 2022;32(5):3142–51. 10.1007/s00330-021-08313-x PMC9038788 34595539

[19] Martin L, et al. Cancer Cachexia in the Age of Obesity: Skeletal Muscle Depletion Is a Powerful Prognostic Factor, Independent of Body Mass Index. J Clin Oncol. 2013;31(12):1539–47. 10.1200/JCO.2012.45.2722 23530101

[20] Faron A, et al. Yttrium-90 radioembolization for hepatocellular carcinoma: Outcome prediction with MRI derived fat-free muscle area. Eur J Radiol. 2020;125:108889. 10.1016/j.ejrad.2020.108889 32087468

[21] Sabel MS, et al. Sarcopenia as a prognostic factor among patients with stage III melanoma. Ann Surg Oncol. 2011;18(13):3579–85. 10.1245/s10434-011-1976-9 21822551

[22] Ohtaka A, et al. Sarcopenia is a poor prognostic factor of castration-resistant prostate cancer treated with docetaxel therapy. Prostate Int. 2019;7(1):9–14. 10.1016/j.prnil.2018.04.002 PMC6424678 30937292

[23] Daly LE, et al. The impact of body composition parameters on ipilimumab toxicity and survival in patients with metastatic melanoma. Br J Cancer. 2017;116(3):310–7. 10.1038/bjc.2016.431 PMC5294486 28072766

[24] Prado CM, et al. Sarcopenia as a determinant of chemotherapy toxicity and time to tumor progression in metastatic breast cancer patients receiving capecitabine treatment. Clin Cancer Res. 2009;15(8):2920–6. 10.1158/1078-0432.CCR-08-2242 19351764

[25] Antoun S, et al. Skeletal muscle density predicts prognosis in patients with metastatic renal cell carcinoma treated with targeted therapies. Cancer. 2013;119(18):3377–84. 10.1002/cncr.28218 23801109

[26] Shachar SS, et al. Prognostic value of sarcopenia in adults with solid tumours: A meta-analysis and systematic review. Eur J Cancer. 2016;57:58–67. 10.1016/j.ejca.2015.12.030 26882087

[27] Luetkens JA, et al. Opportunistic Computed Tomography Imaging for the Assessment of Fatty Muscle Fraction Predicts Outcome in Patients Undergoing Transcatheter Aortic Valve Replacement. Circulation. 2020;141(3):234–6. 10.1161/CIRCULATIONAHA.119.042927 31958246

[28] Papadopoulos E, et al. The impact of sarcopenia on clinical outcomes in men with metastatic castrate-resistant prostate cancer. PLoS ONE. 2023;18(6):e0286381. 10.1371/journal.pone.0286381 PMC10234556 37262068

[29] Iwamoto H, et al. Sarcopenia and Visceral Metastasis at Cabazitaxel Initiation Predict Prognosis in Patients With Castration-resistant Prostate Cancer Receiving Cabazitaxel Chemotherapy. Vivo. 2021;35(3):1703–9. 10.21873/invivo.12430 PMC8193302 33910855

[30] Khan M, et al. 663P Sarcopenia assessment in men with metastatic castration-resistant prostate cancer (mCRPC) undergoing radium 223 (Ra223) therapy. Ann Oncol. 2020;31:S535.

[31] Baracos VE, Arribas L. Sarcopenic obesity: hidden muscle wasting and its impact for survival and complications of cancer therapy. Ann Oncol. 2018;29(suppl2):ii1–9. 10.1093/annonc/mdx810 32169202

[32] Taguchi S, Nakagawa T, Fukuhara H. Inconsistencies in currently used definitions of sarcopenia in oncology. Ann Oncol. 2020;31(2):318–9. 10.1016/j.annonc.2019.10.020 31959350

[33] Lee JH, et al. Prognostic Impact of Sarcopenia in Patients with Metastatic Hormone-Sensitive Prostate Cancer. Cancers. 2021;13(24):6345. 10.3390/cancers13246345 PMC8699789 34944964

[34] De Cecco CN, et al. A noise-optimized virtual monoenergetic reconstruction algorithm improves the diagnostic accuracy of late hepatic arterial phase dual-energy CT for the detection of hypervascular liver lesions. Eur Radiol. 2018;28(8):3393–404. 10.1007/s00330-018-5313-6 29460075

[35] Kan Y, et al. Clinico-radiological characteristic-based machine learning in reducing unnecessary prostate biopsies of PI-RADS 3 lesions with dual validation. Eur Radiol. 2020;30(11):6274–84. 10.1007/s00330-020-06958-8 32524222

[36] Do HM, et al. Augmented Radiologist Workflow Improves Report Value and Saves Time: A Potential Model for Implementation of Artificial Intelligence. Acad Radiol. 2020;27(1):96–105. 10.1016/j.acra.2019.09.014 PMC8189646 31818390

